# Supplementary material for: The Role of Ion Channels and Intracellular Signaling Cascades in the Inhibitory Action of WIN 55,212-2 upon Hyperexcitation
Source: Brain Sci. 2024 Jun 29;14(7):668. doi: 10.3390/brainsci14070668 (PMC11274798; doi:10.3390/brainsci14070668)
Supplement: Supplementary file 1 [file brainsci-14-00668-s001.zip › brainsci-3049523-supplementary.pdf]

Supplementary materials

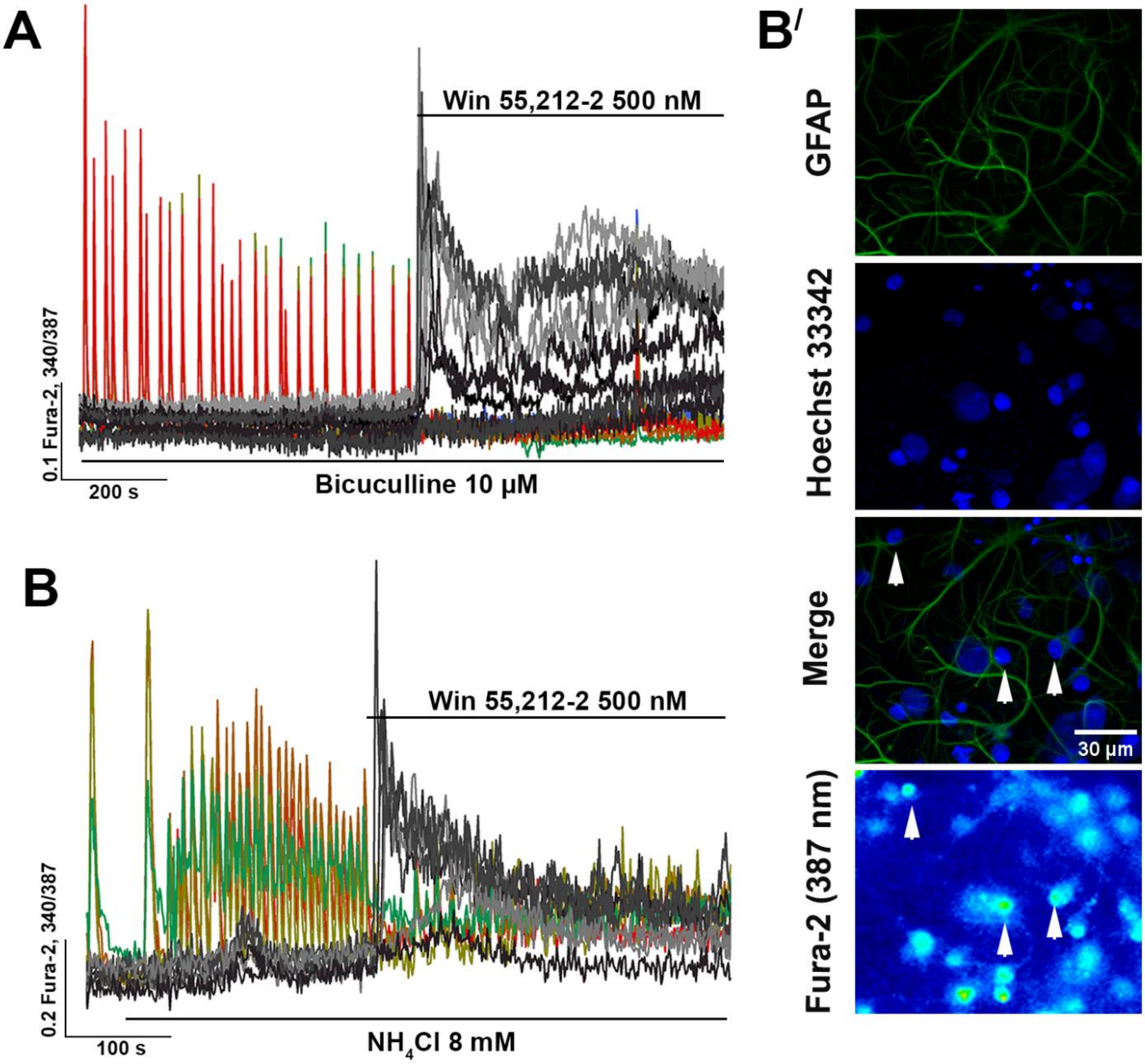

Figure S1. $[Ca^{2+}]_i$ transients in astrocytes (grey curves) occurring in response to WIN 55,212-2 (500 nM) application in the presence of (***A***) bicuculline (10 µM) and (***B***) NH_4Cl (8 mM). Astrocytes were identified using immunostaining (***B′***) with antibodies against specific marker of astrocytes, glial fibrillar acidic protein (GFAP). Nuclei were stained with Hoechst 33342 (5 µg/mL). The image of Fura-2 fluorescence (excitation 387 nm) corresponds to the immunostaining images. White arrows demonstrate the astrocytes responding with $[Ca^{2+}]_i$ elevation to WIN 55,212-2 application in the presence of NH_4Cl (panel ***B***).
